# Efficacy of Subgingival Application of Xanthan-based Chlorhexidine Gel Adjunctive to Full-mouth Root Planing Assessed by Real-time PCR: A Microbiologic and Clinical Study

**DOI:** 10.5681/joddd.2013.017

**Published:** 2013-05-30

**Authors:** Mohammad Taghi Chitsazi, Atabak Kashefimehr, Reza Pourabbas, Adileh Shirmohammadi, Vadood Ghasemi Barghi, Behrouz Daghigh Azar

**Affiliations:** ^1^Associate Professor, Dental and Periodontal Research Center, Tabriz University of Medical Sciences, Tabriz, Iran; ^2^Assistant Professor, Department of Periodontics, Faculty of Dentistry, Tabriz University of Medical Sciences, Tabriz, Iran; ^3^Professor, Dental and Periodontal Research Center, Tabriz University of Medical Sciences, Tabriz, Iran; ^4^Post-graduate Student, Dental and Periodontal Research Center, Tabriz University of Medical Sciences, Tabriz, Iran; ^5^Master of Science, Head of DANESH Molecular Pathology Laboratory

**Keywords:** Xanthan-based chlorhexidine gel, real-time PCR, periodontitis

## Abstract

**Background and aims:**

The aim of this clinical investigation was to compare clinical and microbiological effectiveness of adjunctive CHX gel in the treatment of periodontitis.

**Materials and methods:**

Twenty-four subjects with localized or generalized moderate-to-severe chronic periodontitis underwent scaling and root planing. One tooth in each quadrant with a probing depth of >4 mm was chosen for combined gel and SRP, with contralateral tooth as control (SRP treated site). Clinical assessment was carried out at baseline and 1 and 3 months later; microbial assessment was performed by real-time PCR. Periodontal probing depth (PPD) was considered as primary outcome.

**Results:**

Twenty patients completed the study. Baseline PPDs were 4.90±0.78 and 5.05±0.79 in the SRP and gel groups, respectively (P>0.05), which decreased to 3.67±0.59 and 3.7±0.83 one month after treatment and 3.25±0.65 and 3.38±0.79 three months after treatment. Although values decreased significantly in both groups after one and three months (P=0.001 in the SRP and P=0.001 in the gel group), the inter-group difference was not significant neither at one-month (P=0.47) nor at three-month (P=0.77) intervals. The only clinical parameters exhibiting statistically significant inter-group differences was BOP in both one-month (P=0.004) and three-month (P=0.0001) intervals. All the other clinical measurements showed significant decreases after one and three months in both sites but without inter-group differences.

**Conclusion:**

Subgingival application of xanthan chlorhexidine gel combined with scaling and root planing reduced bleed-ing of periodontal pockets. Clinical trials to evaluate effectiveness of this gel in aggressive and severe periodontitis modified by systemic factors are suggested.

## Introduction

Periodontitis is a chronic inflammatory disease of tooth and supporting structures with clinical signs of bone and connective tissue loss and is mediated by a combination of periodontal pathogens and host defense systems.^1^

Aggregatibacter actinomycetemcomitans (Aa), Porphyromonas gingivalis (Pg), Tannerella forsythensis (Tf) are known as the main pathogens of periodontal disease and treatment of periodontal disease is associated with success in removing and reducing these microorganisms.^2-4^

The main treatment of periodontal disease is nonsurgical treatment by scaling and root planing, which has been confirmed as a gold standard treatment of periodontitis.^5^ However, some patients may not respond favorably to nonsurgical treatment, which might be due to recolonization and reinfection with microorganisms remaining in soft and hard tissues. Other reasons include difficulty of access to deep periodontal pockets, furcation areas and root concavities.^6,7^

In these clinical situations numerous novel approaches have been utilized as mono-therapy or as an adjunct to scaling and root planing in order to improve effectiveness of nonsurgical therapy and achieve long-term periodontal health, especially in patients who do not adequately respond to conventional scaling and root planing.^8,9^ In this field, application of local and systemic antibiotics and different laser systems have been proposed and utilized in recent years.^8,9^ Local delivery of antibiotics has some advantages over systemic use. Local drugs do not have systemic toxicity. Emergence of resistant bacteria is not observed in local forms and also a high concentration of drug remains at the site and induces its therapeutic and antibacterial effect.^10,11^ However, in a systemic review evaluating locally delivered antibiotics, it was concluded that in most cases, in spite of relatively favorable results, improvement in clinical parameters is minor in short term and is not clinically significant.^12^ It has been demonstrated that local administration of CHX is effective in periodontal treatment. Its mechanism of action is through decreasing pellicle formation, alteration of bacterial adhesion to tooth surface and alteration of bacterial cell wall, ultimately leading to cell destruction.^13^ Different forms of CHX have been tested in periodontal treatment. For example, Paoloantonio et al^14^ showed that combined application of controlled release of CHX gel with scaling and root planing results in significant reduction in probing depth. Gupta et al applied xanthan-based chlorhexidine gel and reported significant reductions in probing depth and clinical attachment gain.^15^ Chlosite (Ghimas, Italy) is a gel form material consisting of 0.05% CHX digluconate and 1% CHX and incorporated in a sacharidic cross-linked polymer called xanthan. In contact with water system, it turns into pseudoplastic reticulum which is able to maintain different substances in suspension. This gel undergoes inhibition phenomenon and is removed in 10 to 30 days. CHX gluconate is one of the active materials released during the first day and achieves a concentration of >100 mg/mL which is more than maximum inhibitory concentration of CHX (1 mg/mL) and maintains its bactericidal effect at least for 2 weeks. This product is presented in a prefilled syringe with a blunt tip and a lateral opening that contributes to its safe use without damaging soft tissues.^16^

To the best of our knowledge, limited studies have compared effectiveness of adjunctive CHX gel in the treatment of periodontitis. Therefore, the aim of this clinical and microbial study was to compare the effectiveness of adjunctive CHX gel in the treatment of periodontitis.

## Materials and Methods

### Patient selection

Twenty-four subjects (14 women and 10 men) diagnosed with localized or generalized moderate-to-severe chronic periodontitis participated in the study in the Department of Periodontics, Faculty of Dentistry, Tabriz University of Medical Sciences, Tabriz, Iran. All the subjects gave their informed consent. The patients were enrolled in a longitudinal study, lasting from October 2011 to October 2012. The study design was approved by the Ethics Committee and supported by Tabriz Dental and Periodontal Research Center and also registered in the Iranian Registry of Clinical Trials (IRCT) and allocated the unique code of IRCT2012070210155N1. The nature of this investigation was explained to the participants precisely. The participants with the following inclusion criteria were included in the study: chronic periodontitis, no active periodontal treatment during the previous 6 months, presence of at least one site per quadrant exhibiting pocket depth of ≥4 mm with bleeding on probing, no use of antibiotics for the previous 12 months. The exclusion criteria were: pregnant or lactating women, subjects with any systemic condition influencing the course of periodontal disease or treatment (HIV/AIDS, uncontrolled diabetes) and subjects with any active malignancy of any sort.

### Treatment

Before any nonsurgical treatment, subgingival plaque sampling was carried out. One of the teeth in one quadrant with a probing depth of >4 mm was chosen for plaque sampling. The selected tooth was isolated by sterile cotton rolls and dried gently by air. Supragingival plaque was removed carefully by sterile periodontal curettes. Subsequently, sterile paper point #40 was inserted into the deepest part of the pocket and remained there for 60 seconds. Then the paper point was removed from the pocket and pooled in the transportation vial (Thin Prep-PAP TEST, Hologic, Inc UK) and sent immediately for DNA real-time PCR. Quantification of Porphyromonas gingivalis was carried out by Primer Dedign^TM^Genesig KIT. The fimbbrillinfim A (I) gene which has been identified as a highly specific marker for P. gingivalis was used to detect and quantify P. gingivalis genome.^17^

### DNA extraction

Four µL of internal extraction control DNA were added to each sample in DNA lysis/extraction buffer per sample and completed the extraction according to the manufacturer's protocol.

### Real-time PCR detection

Reaction mix was prepared, including sufficient reactions for the standard curve wells (6 samples in duplicate) and also the negative control. Fifteen μL of this mix were pipetted into each well according to manufacturer's real-time PCR experimental plate set-up. Sample DNA templates for each of the samples (suggested concentration of 5 ng/μL) in RNAse/DNAse free water was prepared. Five μL of diluted DNA template were pipetted into each well, according to experimental plate set-up and standard curve dilution series were prepared. Five μL of standard template were pipetted into each well, according to experimental plate set-up.

### Amplification protocol

Amplification conditions were provided by using Primer Design 2X Precision TM Master Mix.^18^ Measurement of clinical parameters was carried out by a single examiner blinded to the treatment allocation of the patient. Clinical parameters of probing depth (PD), clinical attachment level (CAL), bleeding on probing and gingival recession (REC) were measured at baseline, one month and three months after treatment. O’Leary plaque index was used as a control variable. Positive bleeding on probing was measured based on the presence of bleeding 30 seconds after gentle probing of four sites of each tooth sulcus or pocket and BOP score in these experimental teeth was measured. Quantitative measurement of Porphyromonas gingivalis was carried out at baseline and three months after treatment. All the clinical parameters were measured with manual Williams's periodontal probe (PWD, Hu-Friedy Immunity, USA). Randomization envelope was opened and each tooth in one quadrant was randomized to one of the two treatments: xanthan-based chlorhexidine gel combined with SRP (CHX + SRP) or SRP alone.

Alginate impressions were taken from the upper or lower teeth of patients in order to customize splints. Splints were fabricated precisely to ensure close adaptation with teeth and simplify reproducibility of measurements. In addition, a groove at the site of pockets was made to dictate placement of periodontal probe in the same place during examination period.

In the beginning, all the patients received mechanical nonsurgical therapy by sonic scaler (Varios 350. NSK, Japan). CHX gel was equipped with a special needle with a blunt end and a lateral opening, which was applied in the pocket after the experimental site was isolated by cotton rolls. No additional procedure was carried out on the contralateral tooth. Calibration exercise was performed to obtain acceptable intra-examiner reproducibility for probing depth. Prior to the study and after 3 months, five patients, each with ten teeth with probing depth of >5 mm on at least one aspect of each tooth, were used for calibration. The examiner evaluated the patients on two occasions, 48 hours apart. Calibration was accepted if >90% of the recording could be reproduced within a 1.0-mm difference. The mean of intra-examiner kappa score value was 0.73 for assessment of PD, when PD=5 mm served as the cut-off point.

### Statistical analysis

Because the normality of data was not confirmed by Kolmogorov-Smirnov test, non-parametric test was used to analyze data. Data were analyzed using SPSS 14.0 (SPSS Inc., Chicago, IL, USA). Differences in clinical parameters within groups before and after treatment were evaluated by Friedman test; in addition, Wilcoxon test was used to evaluate differences in pathogens before and after treatment. Mann-Whitney test was used for comparison of clinical and microbiological parameters in each group. Statistical significance was defined at P<0.05.

## Results

From 24 patients enrolled in the study, three patients did not attend the follow-up sessions and were excluded from the study and one patient became pregnant. Twenty patients (11 females and 9 males) with a mean age of 46.5 years completed the study. No adverse reactions from both interventions were reported by the patients.

Probing depth was the primary outcome. According to Table 1, at baseline PPD was 4.90±0.78 in the SRP group and 5.05±0.79 in the gel group (P>0.05). One month after treatment these values decreased to 3.67±0.59 in the SRP group and 3.7±0.83 in the gel group and three month later, to 3.25±0.65 in the SRP group and 3.38±0.79 in the gel group. Although values decreased significantly in both groups after one (P=0.001 in the SRP and P=0.001 in the gel group) and three months (P=0.001 in the SRP and P=0.001 in the gel group) the intergroup difference was not statistically significant neither one month (P=0.47) nor three months (P=0.77) after treatment.

**Table 1:** Clinical assessment of PPD in treated sites

Parameter	Baseline	At 1 month	After 3 months	Difference 0-1 month	p	Difference 0-3 months	p
PPD (mm)							
Gel + SRP	5.05±0.75	3.72±0.83	3.75±0.79	1.32	0.001	1.65	0.001
SRP	4.9±0.78	3.67±0.59	3.25±0.65	1.23	0.001	1.67	0.001
P	0.47	0.77	0.49				

Secondary outcomes were CAL, BOP, REC and microbial evaluation. Clinical measurements of CAL, REC and BOP in treated sites are shown in Table 2. All of these clinical measurements showed significant decreases after one and three months in both treated sites (Table 2). No statistically significant differences were observed in terms of CAL and REC between the two treated sites in both follow-up periods.

**Table 2:** Clinical assessment of CAL, REC, and BOP at treated sites

Parameter	Baseline	At 1 month	After 3 months	Difference 0-1 month	P	Difference 0-3 month	p
CAL (mm)							
Gel + SRP	4.15±0.67	3.82±0.63	3.67±0.65	0.32	0.001	0.47	0.001
SRP	3.9±0.58	3.6±0.50	3.4±0.60	0.3	0.001	0.5	0.001
P	0.166	0.211	0.100				
REC (mm)							
Gel + SRP	-1.075±1.016	0.125±0.39	0.27±0.49	-1.21	0.001	-1.32	0.001
SRP	-0.85±0.56	-0.15±1.22	0.3±1.22	-0.7	0.001	-1.15	0.001
P	0.473	0.624	0.678				
BOP (%)							
Gel + SRP	95±10.25	15±20.51	5±10.2	80	0.001	90	0.001
SRP	90±12.56	31.25±15.9	21.25±9.1	58.75	0.001	68.75	0.001
P	0.173	0.004	0.0001				

**Figure 1:**
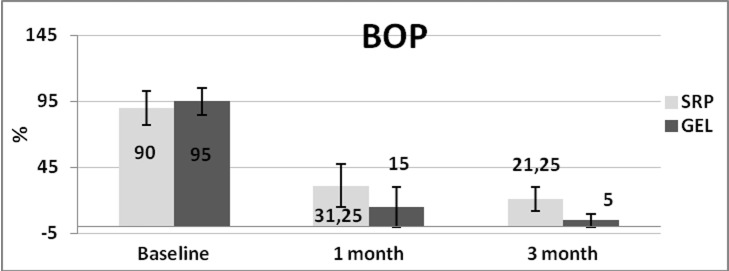
Mean levels of BOP in treated sites at baseline and one month and three months after treatment.

**Figure 2:**
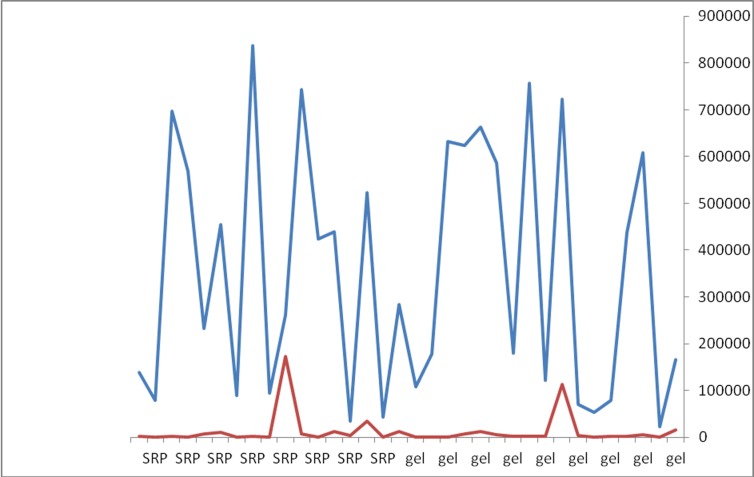
The horizontal of the chart shows P. gingivalis counts. Blue line is baseline measurement andgreen one is the measurement 3 months later. No significant differences were found between the two groups (Mann-Whitney test, P=0.828), although the number of pathogens decreased significantly in both groups in both treated sites after three months (Wilcoxon test, P=0.003, P <0.05).

**Figure 3:**
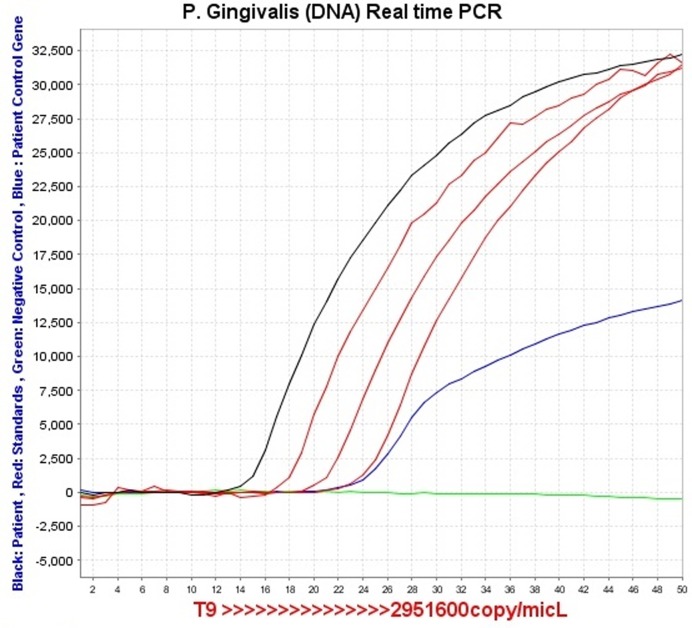
Patient microbial sample analyzed by real-time PCR Primer Design^s^. Black line is related to the quantita-tive number of sample patients. Green line is the negative control. Blue line is the patient control gene.

The only clinical parameters that showed statistically significant intergroup differences were BOP in both one-month (P=0.004) and three-month (P=0.0001) postoperative intervals (Tables 2) (Figures 1–3).

## Discussion

Nonsurgical periodontal therapy in the form of scaling and root planing has been confirmed as a gold standard in periodontal therapy and this treatment is particularly critical in the esthetic zone.^5^

Numerous novel approaches have been utilized as mono-therapy or as an adjunctive to scaling and root planing in order to improve effectiveness of nonsurgical therapy and achieve long-term periodontal health and different results have been reported in this field.^8,9^

Administration of CHX mouthwash has been confirmed as an effective antimicrobial agent and different forms of CHX have been tested in periodontal treatment,^15-17^ including mouthwashes, adhesive gels and chips, with varying results in clinical practice.^14-17,22,23^ This clinical and microbiological study was conducted by monitoring real-time PCR to determine whether topical application of xanthan-based chlorhexidine gel as an adjunctive to scaling and root planing could improve the effectiveness of scaling and root planing. CHX mouthwash has been used successfully with nonsurgical periodontal therapy in plaque-induced gingivitis.^24,25^ On the other hand, subgingival irrigation with CHX or topical application of CHX gel resulted in questionable outcomes supposedly because of inability to reach biologically adequate concentration for sufficient time in periodontal pocket space; therefore, research studies have concentrated on slow-releasing devices with proper substantivity to overcome these limitations of CHX.^25-30^

The results of the current study showed that probing depth decreased significantly in both groups one month and three months after baseline but intergroup differences were not significant. This result is consistent with those of a study by Matesanz et al, in which no significant differences were observed in probing pocket depth between the SRP and xanthan-based gel SRP groups 3 months and 6 months after baseline, although the PPD reduction in the test group was higher than that in the control group.^21^

On the other hand, Paolantonio et al and Gupta et al reported significant decreases in PPD by using xanthan-based chlorhexidine gel as an adjunct to SRP.^14,15^

One of the reasons for insignificant intergroup PPD reductions was good plaque control by the patients. Since plaque index was the control variable in the present study, 4 patients were excluded from the study and the remaining patients had good plaque control and plaque index of less than 10%. Therefore, administration of gel in patients with good plaque control and healthy patients with no systemic disease can be questioned as it could not have additional benefits over SRP. As a result, monitoring the application of CHX gel can be focused on patients with compromised cooperation, such as compromised healthy patients, elderly patients, and also in more severe forms of periodontitis like aggressive periodontitis or periodontitis modified by systemic factors. Good effects of xanthan-based CHX gel is due to its bio-adhesive capability by xanthan and slow release of CHX, which might help maintain acceptable oral hygiene in these patients. In the present study BOP reduction in the gel group was significantly higher than that in the control group one month and three months after treatment. This outcome is consistent with a study by Matesanz, who did not report significant BOP reduction in the gel group between baseline and 3 months after treatment.^21^ This was pointed out in a systematic review by Cosyn^19^ that application of chlorhexidine gel as mono-therapy might reduce bleeding tendency in periodontal pockets but whether it has beneficial effect over SRP is suspected and it is emphasized that more clinical studies be carried out. In spite of the fact that positive BOP is not indicative of periodontal disease, negative BOP is an excellent predictor of periodontal health.^31^ Since BOP positivity in multiple examinations with increasing probing depth is correlated to the progression of periodontitis, controlling gingival inflammation with maintenance of pockets at least in a healthy condition can be a successful treatment option, especially in patients in which periodontal surgery is contraindicated.^32^ The positive result of CHX gel on BOP reduction might provide some advantages to achieve this goal. No adverse reaction was observed due to CHX gel, which is of crucial importance and can facilitate its use in a clinical practice; moreover, it did not exhibit some disadvantages of systemic antibiotics, like the incidence of resistant bacteria or gastrointestinal disturbances.^10,11^

Considering that periodontal disease is caused by disequilibrium between dental plaque and host defense system, monitoring the number of pathogens like P. gingivalis is necessary before and after treatment.^33-35^ Detection of pathogens was influenced by detection methods. Real-time polymerase chain reaction by double fluorescent probes provides precise quantification of bacteria and is one of the most accurate technologies in this field.^36-38^

In the current study P. gingivalis counts decreased significantly after three months with no significant differences between the two groups. In the study by Matesanz et al minor bacterial count reductions were observed one, three and six months after treatment, and intergroup and intragroup differences were not significant.^21^ Bacterial counts in this study were carried out by culture method and the exact quantitative number of cells could not be identified by this method and probable underestimation might have occurred.^37,38^ Therefore, the results of this study cannot be compared with those of the present study, in which real-time PCR quantified the number of bacteria.

In a study by Paoloantonio et al^14^ the percentage of sites contaminated by the microorganisms was determined by conventional PCR. The percentage of positive sites reduced significantly in both groups after 3 and 6 months but intergroup difference was not significant.^14^ Conventional PCR in this study gave endpoint measurements and qualitative measurement was not able to quantify bacteria, which is one of the limitations of conventional PCR compared to real-time PCR which was used in this study.^36-38^

## Conclusion

Within the limitations of the current study, subgingival application of xanthan chlorhexidine gel combined with scaling and root planing may reduce bleeding of periodontal pockets. Other clinical and microbiological parameters were not different between the two groups. It is suggested that clinical trials be designed to evaluate effectiveness of this gel in aggressive periodontitis and severe periodontitis modified by systemic factors.
